# Hemophagocytic Lymphohistiocytosis-Macrophage Activation Syndrome (HLH-MAS) Mimicking Neuropsychiatric Lupus in Systemic Lupus Erythematosus (SLE)

**DOI:** 10.7759/cureus.112940

**Published:** 2026-07-19

**Authors:** Sohira Idrees, Vandan Patel, David Nageh, Lawrence Villanueva, Rehan Shah, Ahmed Naeem

**Affiliations:** 1 Internal Medicine, Touro College of Osteopathic Medicine, Middletown, USA; 2 Internal Medicine, Hudson Regional Hospital, Secaucus, USA

**Keywords:** ferritin-esr dissociation, hemophagocytic lymphohistiocytosis, hscore, hyperferritinemia, macrophage activation syndrome, neuropsychiatric lupus, systemic lupus erythematosus

## Abstract

Hemophagocytic lymphohistiocytosis-macrophage activation syndrome (HLH-MAS) is a life-threatening hyperinflammatory syndrome that can complicate systemic lupus erythematosus (SLE) and present with neurologic manifestations that mimic neuropsychiatric SLE (NPSLE). A 28-year-old woman with a four-year history of SLE initially presented to the emergency department of a community hospital with acute altered mental status and mutism. Laboratory evaluation revealed pancytopenia, markedly elevated ferritin (8,670 ng/mL), hypertriglyceridemia, and hypofibrinogenemia with an erythrocyte sedimentation rate (ESR) that declined from 21 to 6 mm/hr despite active inflammation, a recognized clue distinguishing HLH-MAS from NPSLE. Extensive infectious workup was negative. Although bone marrow biopsy and specialized biomarkers were unavailable at the presenting community hospital, an HScore of 271 (>99% probability of reactive hemophagocytic syndrome) strongly supported the diagnosis of HLH-MAS. High-dose IV methylprednisolone resulted in a progressive decline in ferritin and partial improvement in clinical status, following which the patient was transferred to a tertiary care center for further management. Early recognition of HLH-MAS in SLE patients presenting with acute neurologic symptoms is critical, as misdiagnosis can delay appropriate treatment.

## Introduction

Hemophagocytic lymphohistiocytosis (HLH) can be broadly classified as primary, due to inherited defects in cytotoxic lymphocyte function, or secondary, triggered by infections, malignancy, or autoimmune disease [1,2]. When occurring in the setting of autoimmune disease, it is now increasingly unified under the term hemophagocytic lymphohistiocytosis-macrophage activation syndrome (HLH-MAS) [3].

Among autoimmune conditions, systemic juvenile idiopathic arthritis carries the highest risk of HLH-MAS; however, systemic lupus erythematosus (SLE) is likely an underdiagnosed trigger, with reported prevalence ranging from 0.9% to 4.6% [4] and mortality reported as high as 36% [5]. Diagnosis is particularly challenging when neurologic manifestations predominate, as fever, encephalopathy, seizures, and cytopenias - reported in approximately 10-25% of secondary HLH cases - are also hallmarks of neuropsychiatric SLE (NPSLE), a distinct immune-mediated syndrome of neurologic and psychiatric manifestations in SLE [1]. While their presentations can overlap, HLH-MAS and NPSLE arise from distinct pathophysiologic processes and require different therapeutic approaches, making accurate differentiation critical.

We present a case illustrating the clinical and laboratory features that distinguished HLH-MAS from NPSLE in a young woman with SLE presenting with acute altered mental status.

## Case presentation

A 28-year-old woman with a four-year history of SLE, managed with hydroxychloroquine in an outpatient setting since diagnosis, presented to the emergency department of a community hospital with acute altered mental status and mutism. She had emigrated from Bangladesh, a tuberculosis-endemic region, approximately six months earlier. Three months before presentation, belimumab had been added after she developed progressive weakness, decreased appetite, and alopecia, with subsequent clinical stabilization. However, in the weeks preceding admission, she developed recurrent weakness, dizziness, palpitations, and worsening appetite. Approximately three days before presentation, she developed a fever (maximum reported temperature 101°F), reduced oral intake, and mutism.

On arrival at the emergency department, she was mute but maintained eye contact and appeared severely dehydrated. She was febrile (102.6°F), tachycardic (heart rate (HR): 150 beats/min), tachypneic (respiratory rate (RR): 34 breaths/min), and became progressively hypotensive (blood pressure (BP) declined from 107/64 to 82/51 mmHg; mean arterial pressure (MAP): 61 mmHg). She met systemic inflammatory response syndrome (SIRS) criteria in the setting of immunosuppression, unexplained altered mental status, and concern for sepsis, prompting initiation of empiric vancomycin and piperacillin-tazobactam along with IV fluid resuscitation. Initial laboratory evaluation was notable for pancytopenia, hyponatremia (sodium: 127 mmol/L), elevated aspartate aminotransferase (AST: 147 U/L), and elevated C-reactive protein (CRP: 1.2 mg/dL) with an erythrocyte sedimentation rate (ESR) of 21 mm/hr (Table 1). Urinalysis demonstrated proteinuria (100 mg/dL), trace blood, numerous bacteria, and waxy casts. She was admitted with a working diagnosis of sepsis and altered mental status.

**Table 1:** Trend of key laboratory parameters during hospitalization † IV methylprednisolone was initiated at 500 mg daily on Day 1 and escalated to 1,000 mg daily on Day 2 along with TBO-filgrastim (480 mcg subcutaneous daily); WBC normalization likely reflects granulocyte colony-stimulating factor administration and corticosteroid-induced neutrophil demargination rather than disease improvement. Abbreviations: WBC: white blood cell count; Hgb: hemoglobin; Plt: platelet count; ESR: erythrocyte sedimentation rate; AST: aspartate aminotransferase

Date	WBC (K/µL)	Hgb (g/dL)	Plt (K/µL)	ESR (mm/hr)	AST (U/L)	Ferritin (ng/mL)
ED (Day 0)	1.7	10.0	90	21	147	–
Day 1	1.1	8.7	57	6	96	8,670
Day 2 †	8.2	9.7	60	–	62	6,830
Day 3 †	9.3	8.5	48	–	62	5,360
Day 4 †	7.1	7.8	37	–	50	<1,000
Reference Range	4.5-11.0	12.0-16.0	150-400	0-20	10-40	12-150

**Figure 1:**
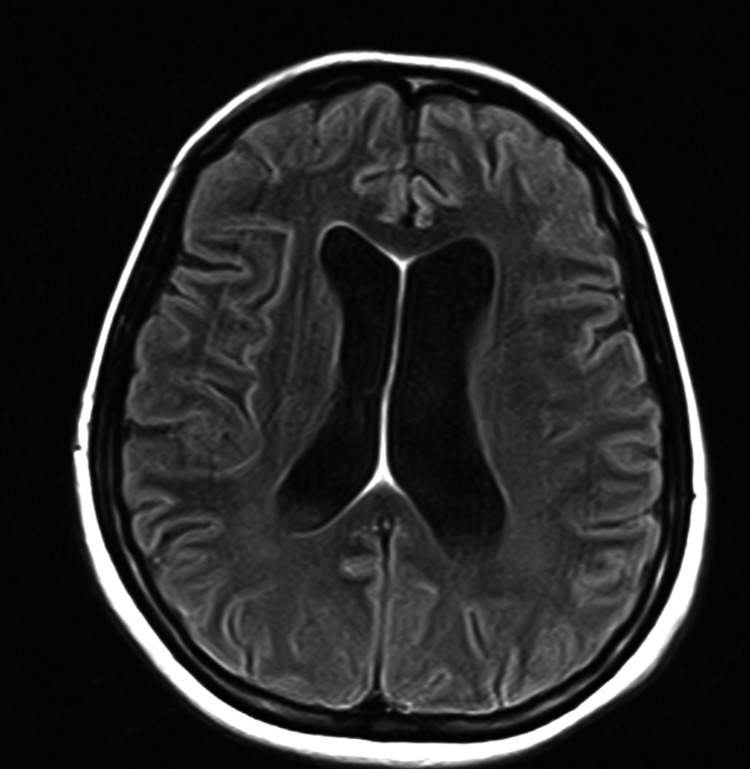
Brain MRI (axial T2 FLAIR) Axial T2 FLAIR MRI of the brain demonstrating ventriculomegaly and minimal periventricular white matter changes consistent with chronic ischemia. No acute infarction, hemorrhage, mass effect, or midline shift. Abbreviations: MRI: magnetic resonance imaging; FLAIR: fluid-attenuated inversion recovery

On hospital Day 1, antibiotics were transitioned from piperacillin-tazobactam to cefepime for broader empiric central nervous system (CNS) coverage, given the patient's unexplained altered mental status, with vancomycin continued. She remained nonverbal but intermittently followed commands, with neurologic examination revealing bilateral upper and lower extremity spasticity and no meningeal signs. CT of the head demonstrated mild ventriculomegaly without acute intracranial abnormality, while CTA of the head and neck was unremarkable. MRI of the brain showed ventriculomegaly with minimal chronic ischemic changes, without acute infarction or hemorrhage (Figure 1). IV methylprednisolone 500 mg daily was initiated for suspected SLE flare. Repeat laboratory testing demonstrated worsening pancytopenia. Ferritin, not obtained in the ED, was markedly elevated at 8,670 ng/mL, and the ESR had declined from 21 to 6 mm/hr despite active inflammation (Table 1). Based on the available data, the differential diagnosis included NPSLE, infectious encephalitis, autoimmune encephalitis, and nonconvulsive seizures. Lumbar puncture was deferred due to thrombocytopenia, and the patient was subsequently transferred to the ICU for continuous video EEG monitoring. 

Over the following three days, vital signs stabilized (temperature: 97-98°F; BP: ~100/60 mmHg; MAP: 73 mmHg). IV methylprednisolone was escalated to 1 g daily. Neurologic examination revealed the patient to be oriented only to name, mute but able to nod, with generalized weakness and persistent spasticity. Continuous video EEG demonstrated seizure-like activity, which resolved with levetiracetam and lacosamide, administered per neurology recommendations.

**Figure 2:**
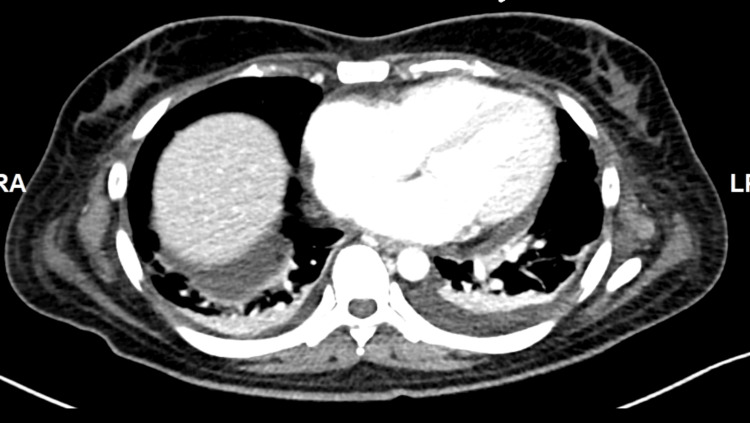
CT chest with contrast (axial) Axial contrast-enhanced computed tomography (CT) of the chest demonstrating bilateral pleural effusions, left greater than right, with increased density at the lung bases consistent with atelectasis; pneumonia cannot be excluded. CT: computed tomography

**Figure 3:**
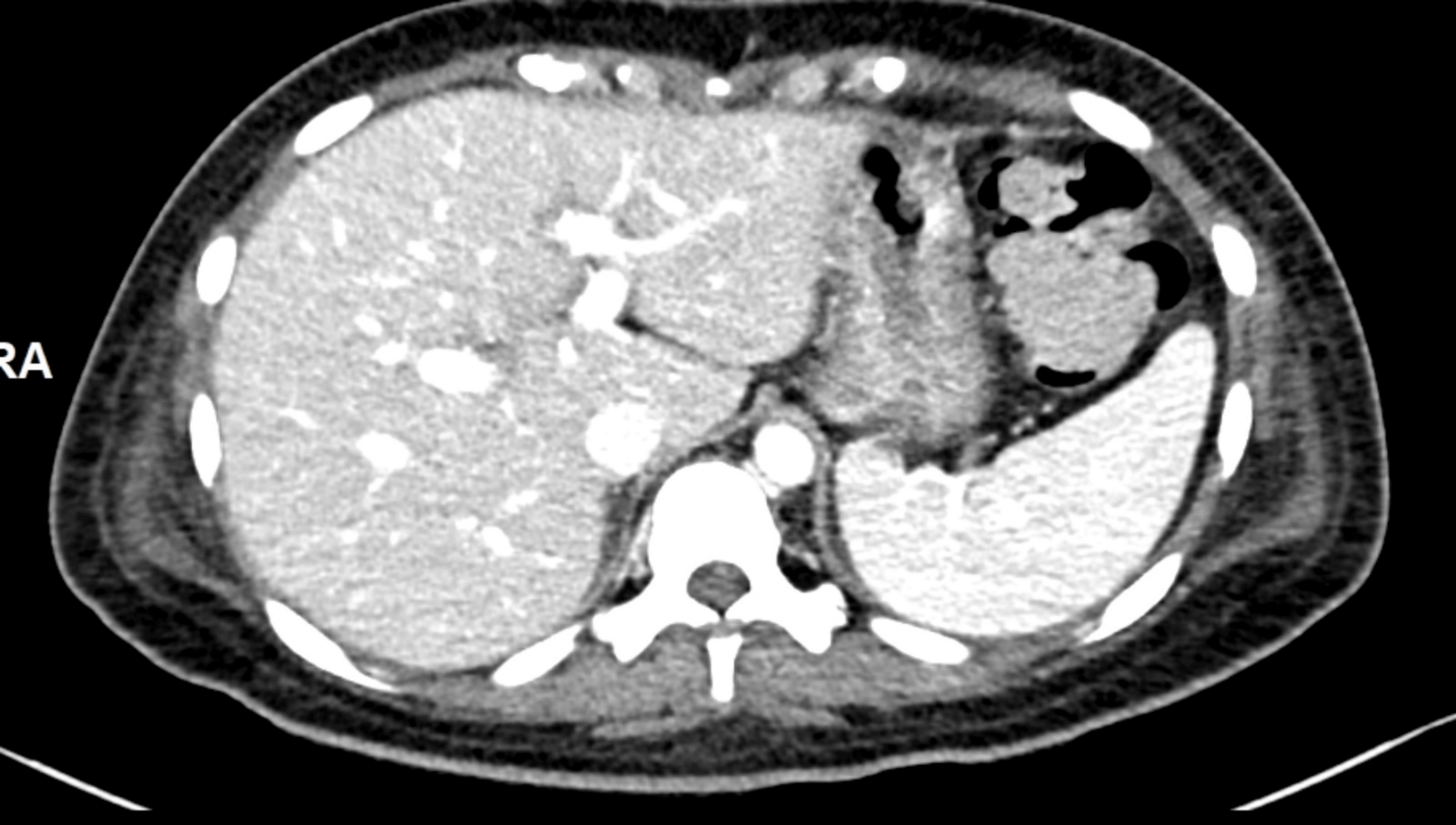
CT abdomen and pelvis with contrast (axial) Axial contrast-enhanced CT of the abdomen and pelvis demonstrating hepatomegaly with possible fatty infiltration and perihepatic ascites. CT: computed tomography

Further laboratory evaluation revealed hypertriglyceridemia (456 mg/dL), hypofibrinogenemia (147 mg/dL), elevated lactate dehydrogenase (LDH: 693 U/L), and decreased complement levels (C3: 30 mg/dL, C4: 12 mg/dL). Anti-dsDNA antibody was 6 IU/mL (indeterminate: 5-9; positive ≥10). Hemoglobin and platelet counts continued to decline, while WBC normalized, likely reflecting TBO-filgrastim administration for neutropenia and the effect of corticosteroids rather than true disease improvement (Table 1, footnote †). CT of the chest demonstrated bilateral pleural effusions, larger on the left, with increased density at the lung bases consistent with atelectasis versus pneumonia; no hilar or mediastinal lymphadenopathy was identified (Figure 2). CT of the abdomen and pelvis revealed hepatomegaly with possible fatty infiltration and ascites (Figure 3). Transthoracic echocardiography demonstrated an ejection fraction of 60% without pericardial effusion.

Extensive infectious workup was negative, including human immunodeficiency virus (HIV), hepatitis B and C, Epstein-Barr virus (EBV) deoxyribonucleic acid (DNA), cytomegalovirus (CMV) DNA, respiratory viral panel, rapid plasma reagin (RPR), and *Mycoplasma pneumoniae*. HSV-1 IgG was positive, consistent with prior exposure; HSV-2 serology was negative. QuantiFERON-TB Gold testing yielded an indeterminate result, likely attributable to profound immunosuppression. Lumbar puncture remained deferred throughout hospitalization due to persistent thrombocytopenia.

The constellation of persistent fever, hyperferritinemia, hypertriglyceridemia, hypofibrinogenemia, progressive cytopenias, declining ESR, and hepatomegaly in the setting of active SLE was consistent with HLH-MAS (Table 2) [2,3]. The HScore was calculated at 271, corresponding to >99% probability of hemophagocytic syndrome (Table 3) [6]. Following high-dose corticosteroid therapy, ferritin declined progressively from 8,670 to <1,000 ng/mL over four days (Table 1). Despite partial clinical improvement, the patient continued to have fluctuating mental status and was transferred to a tertiary care center for consideration of additional immunosuppressive therapy.

**Table 2:** HLH-2004 diagnostic criteria and patient values Diagnostic criteria adapted from Henter et al. [2]. Abbreviations: NK: natural killer; sIL-2R: soluble interleukin-2 receptor; CT: computed tomography; N/A: not assessed; HLH-2004: hemophagocytic lymphohistiocytosis-2004

Criterion	Diagnostic Threshold	Patient Value	Met
Fever	≥38.5°C (101.3°F)	39.2°C (102.6°F)	Yes
Cytopenias (≥2 lineages)	Hemoglobin <9 g/dL, platelets <100 K/µL, or neutrophils <1.0 K/µL	Hemoglobin 7.8 g/dL, platelets 37 K/µL, ANC 0.81 K/µL	Yes
Hypertriglyceridemia and/or hypofibrinogenemia	Triglycerides ≥265 mg/dL and/or fibrinogen ≤150 mg/dL	Triglycerides 456 mg/dL; fibrinogen 147 mg/dL	Yes
Ferritin	≥500 ng/mL	8,670 ng/mL	Yes
Splenomegaly	Clinical or imaging evidence	Not identified on CT abdomen/pelvis	No
Hemophagocytosis	Bone marrow, spleen, liver, or lymph node biopsy	Not assessed	N/A
NK-cell activity	Low or absent	Not assessed	N/A
Soluble CD25 (sIL-2R)	≥2,400 U/mL	Not assessed	N/A

**Table 3:** HScore parameters and calculation Diagnostic HScore parameters and calculation adapted from Fardet et al. [6]. A score of 271 corresponds to a >99% probability of reactive hemophagocytic syndrome [6]. Abbreviations: SLE: systemic lupus erythematosus; CT: computed tomography; WBC: white blood cell count; Hgb: hemoglobin; Plt: platelet count; AST: aspartate aminotransferase

Parameter	Scoring Threshold	Patient Value	Points
Known underlying immunosuppression	HIV-positive or receiving immunosuppressive therapy	SLE on hydroxychloroquine and belimumab	18
Temperature	101.1-102.9°F (38.4-39.4°C)	102.6°F (39.2°C)	33
Organomegaly	Hepatomegaly or splenomegaly	Hepatomegaly on CT	23
Number of cytopenias	3 lineages (Hgb ≤9.2 g/dL, WBC ≤5.0 K/µL, Plt ≤110 K/µL)	Hgb 7.8 g/dL, WBC 1.7 K/µL, Plt 37 K/µL	34
Ferritin (ng/mL)	>6,000	8,670	50
Triglycerides (mg/dL)	>354	456	64
Fibrinogen (mg/dL)	≤250	147	30
AST (U/L)	≥30	147	19
Hemophagocytosis on bone marrow aspirate	Present	Not assessed	0
Total HScore			271

## Discussion

Diagnostic challenge: HLH-MAS vs NPSLE

This case illustrates the difficulty of distinguishing HLH-MAS from NPSLE when neurologic manifestations predominate. In HLH-MAS, CNS injury results from uncontrolled macrophage and T-cell activation, whereas NPSLE is driven by immune complex deposition, complement activation, and antibody-mediated damage [1,3]. Because these mechanisms require fundamentally different therapeutic approaches, accurate differentiation is critical.

The laboratory profile (Table 1) was most consistent with HLH-MAS. Each abnormality is mechanistically linked to the underlying cytokine storm. Macrophage-derived tissue plasminogen activator drives fibrinogen consumption, reducing erythrocyte rouleaux formation and accounting for the paradoxical ESR decline despite ongoing inflammation [1]. This ferritin-ESR dissociation (markedly elevated ferritin with a declining ESR) is a practical bedside diagnostic clue, supported by the 2022 European Alliance of Associations for Rheumatology (EULAR)/American College of Rheumatology (ACR) Points to Consider, which recognizes ESR decline with fibrinogen consumption in HLH-MAS. CRP (1.2 mg/dL), driven by IL-6-mediated hepatic synthesis rather than fibrinogen concentration, was mildly elevated, consistent with inflammation and supporting the interpretation that the ESR decline reflects fibrinogen consumption rather than disease resolution. In contrast, during an uncomplicated SLE flare, ESR typically rises as fibrinogen production remains intact [3].

Macrophage-derived ferritin accounts for the marked hyperferritinemia, which far exceeded the ROC-derived cutoffs reported by Liu et al. (>662.5 ng/mL) and Ammouri et al. (>695 ng/mL) for discriminating SLE-associated MAS from uncomplicated SLE flare [7,8]. Cytokine-mediated suppression of lipoprotein lipase contributes to the hypertriglyceridemia, while elevated LDH reflects hemophagocytosis-driven RBC destruction and hepatocyte injury from lymphohistiocytic infiltration [1].

These findings are not characteristic of NPSLE, in which fibrinogen is typically normal or elevated, ESR is correspondingly elevated, and ferritin is only mildly increased. Additional findings argued against NPSLE as the primary diagnosis: brain MRI showed only minimal chronic ischemic changes without the focal white matter hyperintensities or vasculitic changes more typical of NPSLE, while anti-dsDNA antibody was 6 IU/mL (indeterminate, 5-9; positive ≥10), rather than elevated (Table 4).

**Table 4:** Distinguishing features: HLH-MAS versus neuropsychiatric SLE Data adapted from Henter [1] and Shakoory et al. [3]. Abbreviations: ESR: erythrocyte sedimentation rate; LPL: lipoprotein lipase; CNS: central nervous system; MRI: magnetic resonance imaging; dsDNA: double-stranded DNA; HLH-MAS: hemophagocytic lymphohistiocytosis-macrophage activation syndrome; SLE: systemic lupus erythematosus

Parameter	HLH-MAS	Neuropsychiatric SLE	Patient Value
Pathophysiology	Cytokine storm (macrophage/T-cell activation)	Immune complex-mediated CNS inflammation; autoantibody-mediated neuronal injury	—
Typical presentation	Fever, cytopenias, multiorgan inflammation	Seizures, psychosis, cognitive dysfunction, headache	Fever, pancytopenia, encephalopathy, seizures, hepatomegaly
Ferritin (ng/mL)	↑↑ (often >2,000; macrophage-derived)	Normal to mild ↑	8,670 (↑↑)
ESR (mm/hr)	↓ (fibrinogen consumption)	↑ (acute-phase response)	21 → 6 (↓)
Fibrinogen (mg/dL)	↓ (consumptive coagulopathy)	Normal to ↑	147 (↓)
Triglycerides (mg/dL)	↑ (due to ↓ LPL)	Usually normal	456 (↑)
Cytopenias	Common; progressive, multilineage (marrow hemophagocytosis)	Variable, typically mild	Progressive pancytopenia
Liver enzymes	↑ (lymphohistiocytic infiltration)	Mild ↑ possible	AST 147 U/L (↑)
Brain MRI	Often normal/nonspecific	Focal white matter lesions, atrophy, vasculitis	Minimal chronic ischemic changes
Anti-dsDNA antibody (IU/mL)	Variable	Typically elevated	6 (indeterminate; positive ≥10)

Pathogenesis: a multi-hit model

This case likely reflects a multifactorial process consistent with the threshold model of secondary HLH, in which baseline immune dysregulation from active SLE, immune modulation with belimumab, and a potential unrecognized infectious trigger may have collectively lowered the threshold for uncontrolled cytokine storm [1].

Decreased complement levels support active SLE as the most probable primary driver, consistent with multicenter data identifying active SLE as the trigger in up to 65% of SLE-associated MAS cases [3,7]. Belimumab, initiated approximately three months prior, warrants consideration as a contributing factor. As a BLyS (BAFF) inhibitor, it primarily targets B-cell survival rather than the cytotoxic T-cell and NK-cell pathways that drive HLH-MAS; thus, it is unlikely to have directly caused HLH-MAS. Although no published reports have directly identified belimumab as a trigger for HLH-MAS, its immunomodulatory effects may have facilitated a permissive immune environment for uncontrolled cytokine activation.

The patient's recent immigration from a tuberculosis-endemic region introduces the possibility of an unrecognized infectious "second hit." Although she had no respiratory symptoms at presentation, CT chest revealed bilateral pleural effusions and basilar opacities without hilar or mediastinal lymphadenopathy - findings that are nonspecific but may be seen with active SLE, HLH-MAS, or other inflammatory and infectious processes, including tuberculosis [9,10]. No sputum acid-fast bacilli (AFB) smear/polymerase chain reaction (PCR), pleural fluid studies, cultures, or biopsy were obtained to further evaluate these findings. The indeterminate QuantiFERON-TB Gold result, an expected limitation in active SLE with lymphopenia, does not exclude latent or active infection.

While many SLE flares involve immune activation, progression to HLH-MAS likely requires an additional failure of cytotoxic immune regulation, consistent with loss of physiologic control of macrophage activation [1].

Treatment considerations

Early escalation beyond corticosteroids is critical in HLH-MAS, as delayed treatment is associated not only with significant mortality but also with risk of irreversible neurologic injury when CNS involvement is present [1,3]. In this patient, high-dose IV methylprednisolone produced a progressive decline in ferritin over four days (Table 1). For HLH-MAS, cyclosporine and anakinra are established adjuncts to glucocorticoids, and IVIG may provide additional immunomodulation without significant immunosuppression [1,3]. In patients with refractory disease despite these measures, etoposide can be considered, though evidence is less favorable in autoimmune-associated HLH-MAS than in EBV-associated HLH [3,11,12]. These therapies were unavailable at the presenting community hospital, necessitating transfer to a tertiary care center.

Limitations

Bone marrow biopsy, soluble CD25, and NK-cell activity were not available at the presenting facility. Lumbar puncture remained deferred throughout hospitalization due to persistent thrombocytopenia, precluding CSF analysis. The patient met 4 of 5 assessable HLH-2004 criteria (fever, cytopenias, hypertriglyceridemia/hypofibrinogenemia, and hyperferritinemia); hemophagocytosis, NK-cell activity, and soluble CD25 were not assessed, rather than the traditional threshold of 5 of 8 [2]. However, recent evidence, including a 2025 multicenter validation across 13 cohorts by Lachmann et al. and a 2025 New England Journal of Medicine (NEJM) review, supports fulfillment of 4 criteria as providing optimal diagnostic accuracy for secondary HLH in adults [1,13]. Additionally, ESR was measured only on Days 0 and 1. Although subsequent ESR measurements were not obtained, the observed 71% decline was consistent with concurrent hypofibrinogenemia. The HScore of 271, corresponding to >99% probability of hemophagocytic syndrome, provided additional diagnostic confidence [6]. Long-term follow-up data were not available, as care was transitioned at the time of transfer.

## Conclusions

HLH-MAS should be considered in SLE patients presenting with acute altered mental status and laboratory evidence of systemic hyperinflammation. The ferritin-ESR dissociation, while requiring validation in larger cohorts, may serve as a useful bedside clue for distinguishing HLH-MAS from an active SLE flare, and the HScore can support early recognition in settings where specialized diagnostics are unavailable. This case further illustrates the clinical utility of the threshold model in understanding HLH-MAS pathogenesis in the setting of multiple converging contributors. Early recognition and prompt escalation of immunosuppressive therapy, when appropriate for the underlying etiology, remain essential to reduce mortality and prevent irreversible neurologic sequelae.
